# Genome-Wide Association Study Uncovers Genomic Regions Associated with Coleoptile Length in a Worldwide Collection of Oat

**DOI:** 10.3390/genes15040411

**Published:** 2024-03-26

**Authors:** Pingping Zhou, Yuankun Liu, Mengxian Yang, Honghai Yan

**Affiliations:** College of Agronomy and Biotechnology, Yunnan Agricultural University, Kunming 650201, China; zhoupingping89@163.com (P.Z.);

**Keywords:** oat, coleoptile length, GWAS, deep sowing, genetic variation

## Abstract

The length of coleoptile is crucial for determining the sowing depth of oats in low-precipitation regions, which is significant for oat breeding programs. In this study, a diverse panel of 243 oat accessions was used to explore coleoptile length in two independent experiments. The panel exhibited significant variation in coleoptile length, ranging from 4.66 to 8.76 cm. Accessions from Africa, America, and the Mediterranean region displayed longer coleoptile lengths than those from Asia and Europe. Genome-wide association studies (GWASs) using 26,196 SNPs identified 34 SNPs, representing 32 quantitative trait loci (QTLs) significantly associated with coleoptile length. Among these QTLs, six were consistently detected in both experiments, explaining 6.43% to 10.07% of the phenotypic variation. The favorable alleles at these stable loci additively increased coleoptile length, offering insights for pyramid breeding. Gene Ontology (GO) analysis of the 350 candidate genes underlying the six stable QTLs revealed significant enrichment in cell development-related processes. Several phytochrome-related genes, including auxin transporter-like protein 1 and cytochrome P450 proteins, were found within these QTLs. Further validation of these loci will enhance our understanding of coleoptile length regulation. This study provides new insights into the genetic architecture of coleoptile length in oats.

## 1. Introduction

Common oat (*Avena sativa* L., 2n = 6x = 42, AACCDD) is globally recognized as the sixth most important cereal crop, following wheat, maize, rice, barley, and sorghum [1]. In comparison to other temperate cereal crops such as wheat and barley, oats have gained a reputation for their superior adaptability to marginal environments [2]. This attribute positions oats as a crucial grain crop for communities residing in marginal ecologies throughout the developing world [3]. Presently, the major oat growing regions are primarily concentrated between latitudes 40° and 60° N in America and Europe, with a smaller proportion of global oat production originating from the southern hemisphere, e.g., Australia and New Zealand [4]. Most oat production areas face arid or semi-arid climates, which leads to insufficient topsoil moisture for seed germination. In such cases, deep sowing is required to access enough moisture in the soil for seed germination. For instance, oats grown in the low-water supply areas of New Zealand are sown at a depth of more than 5 cm to protect seeds from dehydration caused by high rates of evaporation [5]. Deep sowing could also reduce seed damage from animals and pests [6] and protect the seedlings from pre-emergent herbicides [7].

Despite its merits, deep sowing is strongly associated with a significant decrease in seedling emergence, while it is a prerequisite for achieving optimal crop yields [8,9,10]. In cereal crops, the maximum potential sowing depth is largely determined by the length of the coleoptile. The coleoptile is a protective sheath-like tissue that covers the primary leaf of germinating seedlings of monocotyledons, safeguarding the emerging shoots as they break through the soil to the surface [11]. In crops such as wheat and barley, varieties with longer coleoptile lengths have shown improved seedling emergence and grain yield when sown at greater depths [12,13]. Consequently, breeding programs in water-deficit regions focus on developing cultivars with long coleoptiles as one of their main breeding goals.

Considerable studies have been carried out to evaluate the coleoptile length in major cereal crops, such as rice [14], wheat [11,15,16], and barley [17,18], revealing a wide range of variation in these crops. In contrast, there is limited information available on the coleoptile length of oat cultivars. Kaufmann [19] investigated the coleoptile length of four oat varieties planted at different seeding depths and found that these oat varieties exhibited similar coleoptile lengths. In another study, Koçak et al. [20] assessed the coleoptile length of 167 Turkish oats and revealed a wide spectrum of lengths, ranging from 12.33 to 45.00 mm. Furthermore, Radford and Key [5] evaluated the coleoptile length of 16 diverse oat genotypes at various temperatures and found that the optimal temperature for maximum coleoptile length was 15 °C. However, due to the limited number or diversity of oat germplasms used in these studies, further investigations are necessary to fully understand the extent of variation in coleoptile length in oats.

Studies conducted in other crops have demonstrated that coleoptile length is controlled by multiple genes with high heritability and significant additive effects [11,15,21], indicating the possibility of enhancing coleoptile length through genetic improvement. Extensive efforts have been dedicated to identifying quantitative trait loci (QTLs) associated with coleoptile length in rice [22,23,24], wheat [25,26,27,28,29], and barley [30,31] using bi-parental and/or multi-parental mapping populations. These studies have successfully identified numerous QTLs distributed on almost all chromosomes. Furthermore, genome-wide association studies (GWASs) based on linkage disequilibrium have also been employed to detect markers/QTLs associated with coleoptile length in these crops [11,15,18]. These GWASs not only validated previously identified QTLs from linkage mapping but have also discovered novel ones, thereby providing valuable insights for breeding and genomic research in these crops.

Compared to major crops, the progress in elucidating the genetic control of coleoptile length in oats has been significantly slower, primarily due to the scarcity of molecular markers [32,33]. Recent advancements in oat genomics have led to the development of high-quality and fully annotated oat genomes [34,35], offering promising prospects for oat genomic studies. For example, by utilizing genotyping-by-sequencing (GBS) data from 659 oat accessions and aligning them to the reference genome of the hulless oat cultivar ‘Sanfensan’, we successfully identified 49,702 high-quality SNPs [34]. These SNPs subsequently facilitated the detection of markers associated with hulless grain [34], groat protein content [36], and grain size [33]. Despite the importance of coleoptile length as a trait in oats, very few studies have been conducted to identify the genomic regions associated with this characteristic. Only one previous study identified five markers distributed on five oat chromosomes that were associated with coleoptile length [20]. Due to the complexity of coleoptile length, further identification of additional QTLs and markers is necessary. Therefore, in this study, we conducted a comprehensive genome-wide association study using a repertoire of over 26,000 genetic markers to delineate the marker–trait associations (MTAs) for coleoptile length. To achieve this, we employed a subset of 243 oat accessions from the larger collection of 659 oat lines. This subset covered a wide range of genetic diversity, consisting of 70 modern cultivars and 173 landraces. The primary objectives of this study included (i) evaluating the genetic variation in coleoptile length within the diverse oat genotypes, (ii) identifying the genomic loci linked to this trait through GWAS, and (iii) characterizing the most plausible candidate genes underlying the detected MTAs. The findings of this study will enhance our comprehension of the genetic foundation of coleoptile length and aid in the creation of genetic tools for cultivating long coleoptiles that are suitable for deep sowing in regions with low precipitation.

## 2. Materials and Methods

### 2.1. Plant Materials

In a previous study, a diverse oat panel (referred to as the Diverse Oat Panel) consisting of 659 germplasms collected from more than 50 countries was genotyped by GBS. Principal component analysis (PCA) revealed a weak population structure in the Diverse Oat Panel [34]. Based on the results of the PCA, a subset of the Diverse Oat Panel comprising 243 oat accessions was selected for phenotyping coleoptile length in this study. This subset included 70 modern cultivars and 173 landraces and was chosen to represent most of the genetic diversity and geographical distribution within the Diverse Oat Panel (Table S1, Figure S1).

### 2.2. Phenotype Evaluation

The evaluation of coleoptile length in this study followed a blotting-paper germination approach, as described in a previous study with some minor modifications [15]. In brief, five seeds with uniform size and plumpness were selected from each oat accession and subjected to a cleaning process involving 3% H_2_O_2_ and distilled water. Subsequently, the seeds were arranged in a line with a spacing of 1 cm between each seed on a soaked germination towel (5 cm × 5 cm) placed on a sheet of wax paper. The towel and wax paper were then loosely rolled and secured with a rubber band, forming a vertical arrangement inside a plastic box. To ensure optimal conditions for germination, the plastic boxes were covered with an opaque plastic bag to exclude light and minimize water loss through evaporation. The boxes were then placed in a refrigerator at 4 °C for 24 h to break seed dormancy. Following the dormancy-breaking period, the seedlings were allowed to elongate in a growth chamber at a constant temperature of 15 °C for a period of 10 days. Coleoptile length was measured by determining the distance from the coleoptile node to the tip of the coleoptile, following the methodology outlined by Radford and Key [5]. The experimental design utilized a completely randomized block design with three replicates, and the experiment was repeated once to ensure the robustness and reliability of the results.

### 2.3. Statistical Analysis

Analysis of variance (ANOVA) was conducted using the general linear model (GLM) procedure, with genotypes and experiments treated as fixed variables and replication within the experiment as a random variable. To assess the consistency among replicates within each experiment and between the two independent experiments, Pearson’s correlation coefficients were calculated based on the mean values using the “corrplot” package (v0.92) available at https://github.com/taiyun/corrplot (accessed on 10 January 2024). The broad-sense heritability was calculated as the ratio of total genetic variance to total phenotypic variance [37]. A two-tailed Student’s *t*-test was used to estimate the significance of the differences in coleoptile length among different groups.

### 2.4. Genomic Data Analysis

The association panel utilized in this study has been previously genotyped with a set of 49,702 high-quality SNPs derived from GBS data [34]. The reference genome “Sanfensan” was used to assign the physical positions of these SNPs. To ensure the quality of the data, the SNP dataset underwent several filtration steps. Loci with missing data exceeding 20%, minor allele frequency (MAF) below 0.05, and heterozygosity above 10% were excluded. After filtration, the dataset was left with 26,196 SNPs. To impute missing genotypes, the Linkage Disequilibrium K-Number Neighbor Imputation method implemented in the TASSEL software (v5.0) was employed, utilizing information from the ten nearest neighbors [38]. The resulting genotypic matrix was then utilized for the GWAS analyses.

### 2.5. Association Analysis

For each experiment, GWAS was conducted independently using TASSEL 5.0. Two models were compared: the general linear model (GLM) incorporating population structure (Q) and the mixed linear model (MLM) incorporating both population structure (Q) and familial relatedness (K). Principal component analysis revealed that the first five principal components (PCs) accounted for 50% of the phenotypic variation, which was considered sufficient for correcting for stratification and was utilized as a correction for population structure. Familial relatedness was estimated using a centered identity-by-state method implemented in TASSEL 5.0. The *R*^2^ values generated in the GLM or MLM statistics output were used to estimate the proportion of phenotypic variability explained by each significant marker. Manhattan and quantile–quantile (QQ) plots were generated using the R package “CMplot” [39]. Initially, a Bonferroni correction was applied, setting a significance threshold of *p* = 3.82 × 10^−5^ (1/26,196) to identify significant associations between SNP markers and coleoptile length. However, this threshold was deemed overly conservative. Therefore, a less stringent *p*-value (0.001), as used in previous studies [40], was employed to identify significant marker–trait associations (MTAs). For robustness, only the MTAs that were significant in both experiments were further analyzed.

### 2.6. Comparative Mapping

Five SNPs associated with coleoptile length in oats were identified in a previous study [20]. To compare the results of this study with ours, the DNA sequences of these markers were aligned to the “Sanfensan” reference genome. The best hits with a query cover >95% and an e-value < 1 × 10^−20^ were considered chromosomal locations of the associated markers within the reference genome.

### 2.7. Effects of Favorable Alleles

The effect of the favorable allele was determined using the following formula: [Mean (fav) − Mean (unf)]/Mean (unf) × 100, where Mean (fav) represents the group means of homozygous groups with favorable alleles, while Mean (unf) represents the group means of homozygous groups with unfavorable alleles. The favorable alleles were defined as those with positive effects, leading to longer coleoptile length. Conversely, the alternative alleles with negative effects that lead to shorter coleoptile length were considered unfavorable alleles. The significance of the difference in coleoptile length between accessions with favorable alleles and unfavorable alleles was estimated by using a two-tailed Student’s *t*-test. A linear regression analysis was conducted to estimate the relationship between the number of favorable alleles and coleoptile length.

### 2.8. Putative Candidate Gene Analysis

To identify potential candidate genes, we utilized the annotation file of the “Sanfensan” reference genome. This allowed us to extract high-confidence genes located within a 2.29 Mb flanking the LD region surrounding significant SNPs included in stable QTLs from the GWAS. To annotate these potential candidate genes, Gene Ontology (GO) annotation of the potential candidate genes was performed using eggNOG-mapper v2 [41]. GO enrichment analysis was conducted using the clusterProfiler package v4.0 [42], with a threshold of a Benjamini–Hochberg corrected *p*-value (Q value) of ≤0.05 to determine significantly enriched GO terms.

## 3. Results

### 3.1. Coleoptile Length in Oat Accession

**Figure 1:**
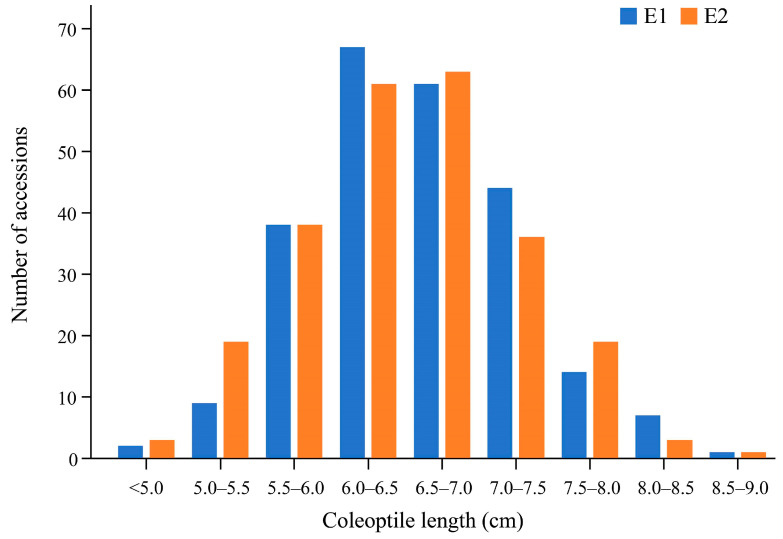
Distribution of mean coleoptile lengths of the association panel in two independent experiments. E1, Experiment 1; E2, Experiment 2.

This study investigated the coleoptile length of 243 oat accessions collected from 54 countries in two separate experiments (Table S1). The ANOVA revealed significant variation among different genotypes and experiments (Table 1). The coleoptile length ranged from 4.70 to 8.76 cm for Experiment 1 (E1) and from 4.66 to 8.68 cm for E2, showing a 1.86- and 1.86-fold variation in both cases (Figure 1, Table 2). Most oat accessions, i.e., 86.42% and 81.48%, had coleoptile lengths ranging from 5.5 to 7.5 cm. Only 22 and 23 accessions displayed a coleoptile length exceeding 7.5 cm in E1 and E2, respectively (Figure 1). These germplasms could serve as valuable genetic resources for future improvements in coleoptile length. A significant correlation was observed between coleoptile length in the two independent experiments (*R* = 0.91, *p* < 0.001; see Figure S2) and the estimated broad-sense heritability (*H*^2^) of coleoptile was 0.86 (Table 2), indicating a high heritability.

**Figure 2:**
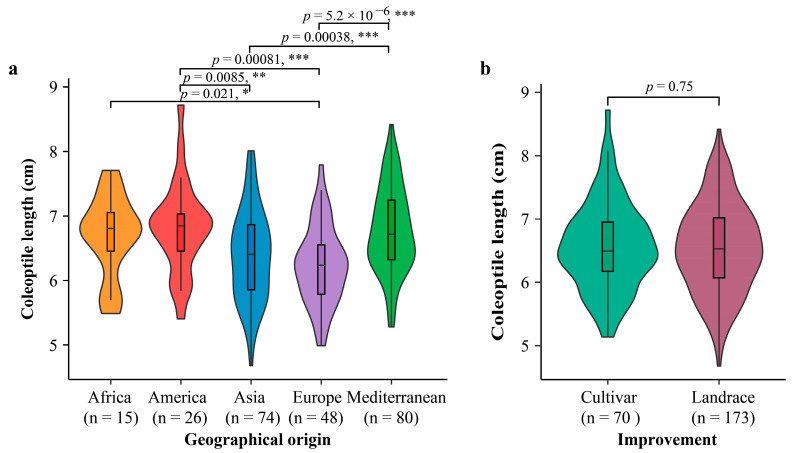
Comparison of coleoptile length of accessions from different origins (**a**) and between cultivars and landraces (**b**). The coleoptile length was averaged from the two independent experiments. * *p* < 0.05; ** *p* < 0.01; *** *p* < 0.001.

We subsequently conducted a comparison of the mean coleoptile length among oat accessions originating from different geographic origins. To represent the coleoptile length of the varieties, we used the average data from the two experiments, as they exhibited a strong correlation. The mean coleoptile lengths for accessions from Africa, America, Asia, Europe, and the Mediterranean were 6.69, 6.82, 6.38, 6.23, and 6.79 cm, respectively. Notably, accessions from America and the Mediterranean displayed significantly longer coleoptiles compared to those from other regions (Figure 2). Conversely, accessions from Europe exhibited the shortest mean coleoptile lengths. There was no significant difference in mean coleoptile length observed between cultivars (6.57 cm) and landraces (6.54 cm). In conclusion, our findings revealed a substantial variation in coleoptile length within the oat germplasms, with a stronger correlation observed between coleoptile length and the breeding origins rather than the improvement status.

### 3.2. Genome-Wide Association Analysis of Coleoptile Length

**Figure 3:**
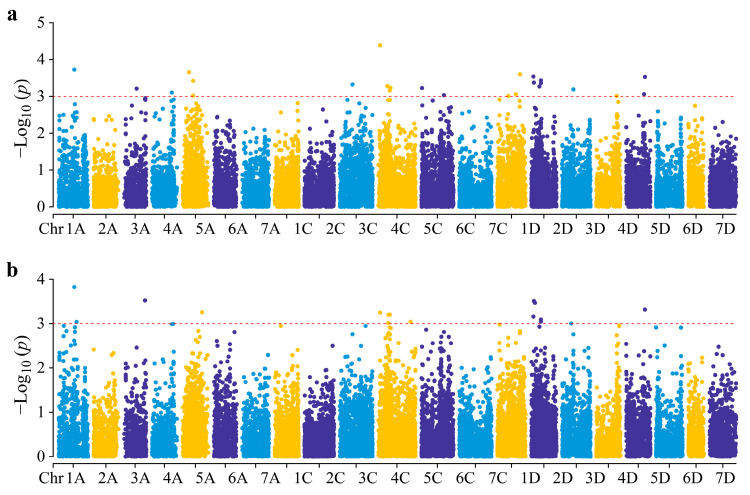
Manhattan plots for coleoptile length. (**a**) E1. (**b**) E2.

Two models, GLM with Q and MLM with Q + K, were utilized for GWAS analyses. Comparison of the quantile–quantile (Q-Q) plots from GLM and MLM revealed that the MLM model generated an observed-to-expected *p*-value relationship closely aligned with expectations under the null hypothesis (Figure S3), suggesting its appropriateness for this study. GWAS analyses identified 25 and 16 significant SNPs (−log_10_ *p* > 3) associated with coleoptile length in E1 and E2, respectively, explaining a phenotypic variation ranging from 6.30 to 10.07% (Figure 3, Table S2). These SNPs were distributed across 12 oat chromosomes. Based on our previous study indicating an LD decay of approximately 2.29 Mb [34], significant markers within a 2.29 Mb region were considered representative of a single QTL. In total, 32 QTLs for coleoptile length were detected, and they were not asymmetrically distributed. The highest number of QTLs (8) was detected in 4C, followed by 1D (5). Six QTLs, one on each of chromosomes 1A, 4C, and 4D, and three on chromosome 1D, were consistently detected in both experiments and explained a significant portion (6.43–10.07%) of the observed phenotypic variations (Table 3).

### 3.3. Comparison to Previous Studies

**Figure 4:**
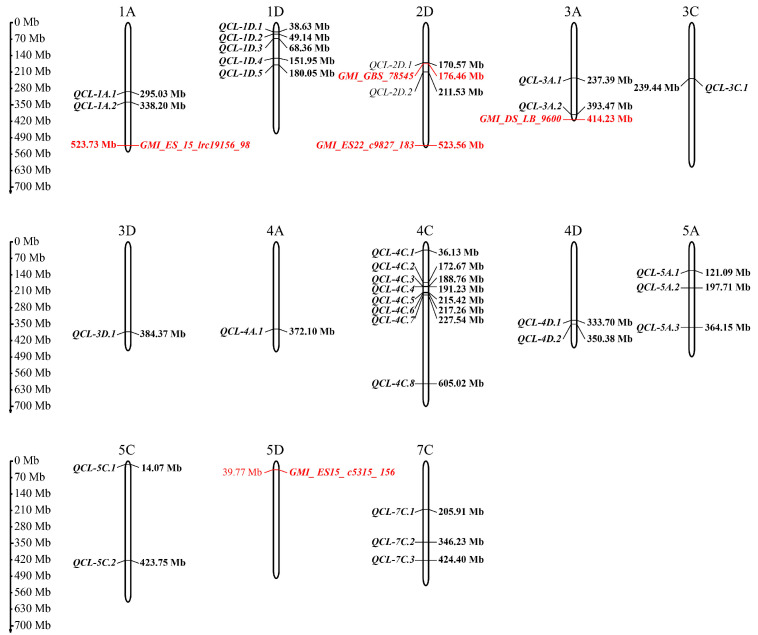
The chromosomal locations of QTLs associated with coleoptile length in oat identified in this study and a previous study [20]. QTLs identified by Koçak et al. [20] are highlighted in red.

The chromosomal positions of previously reported SNPs associated with coleoptile length [20] were determined by aligning their DNA sequences to the “Sanfensan” reference genome. It was found that all five reported SNPs were unambiguously located on specific chromosomes, as shown in Figure 4. A comparison was conducted between the 32 QTLs in this study and these known SNPs, revealing that one of the QTLs identified in this study overlapped with these known loci. Therefore, all of the QTLs identified in this study are independent and distinct from the previously reported SNPs associated with coleoptile length.

### 3.4. Effects of Favorable Allele on Coleoptile Length

**Figure 5:**
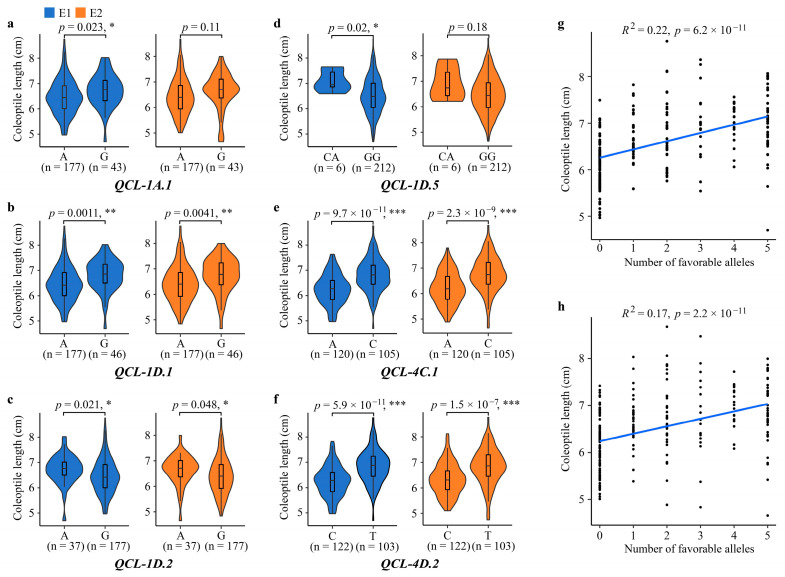
The allelic effects of the six stable QTLs on coleoptile length. (**a**–**f**), Allelic effects of each individual QTL on coleoptile length. (**g**,**h**), Linear regression between the number of favorable alleles and coleoptile length in E1 (**g**) and E2 (**h**). Two-tailed Student’s *t*-test was used to determine the significant differences between mean values of two alleles in (**a**–**f**). *, significant at *p* < 0.05; **, significant at *p* < 0.01, ***, significant at *p* < 0.001.

To investigate the impact of favorable alleles on coleoptile length, the coleoptile length was calculated for the two alleles of each stable QTL. The presence of favorable alleles increased coleoptile length by 4.02 to 9.26% in E1 and by 3.09 to 8.79% in E2 (Table 3, Figure 5a–f). The number of favorable alleles per accession ranged from 0 to 5, with an average of 1.39 across the six loci in this association panel. The accessions were categorized based on the number of favorable alleles, and the average coleoptile length for each group was analyzed to assess the effects of the number of favorable alleles on coleoptile length. In both experiments, a significant linear relationship was observed between the number of favorable alleles and coleoptile length (E1: *R*^2^ = 0.22, *p* < 0.001; E2: *R*^2^ = 0.16, *p* < 0.001) (Figure 5g,h), indicating that an increased number of favorable alleles is associated with enhanced coleoptile length.

### 3.5. Identification of Putative Candidate Genes

Candidate genes were identified by considering genes located within a 2.29 Mb LD block on each side of the six QTLs stably associated with coleoptile length. Through screening the annotated genes in the “Sanfensan” reference genome, a total of 350 potential candidate genes were identified (Table S3). To gain insights into their functional roles, GO enrichment analysis was performed on these candidate genes. This analysis classified most of these genes into three categories: biological process (BP), cellular component (CC), and molecular function (MF). A total of twenty-eight significantly enriched GO terms (Q value < 0.05) were identified, including twenty-two BPs and six MFs (Table S4, Figure S4). The significantly enriched BPs were primarily related to cell growth. Notably, one gene (*A.satnudSFS4D01G002954* in QTL *QCL-4D.2*) encoding auxin transporter protein 1 in and five genes (*A.satnudSFS1A01G005205*, *A.satnudSFS1A01G005149*, and *A.satnudSFS1A01G005147* in QTL *QCL-1A.1*, *A.satnudSFS4C01G001030* in QTL *QCL-4C.1*, and *A.satnudSFS4D01G002963* in QTL *QCL-4D.2*) predicted to encode cytochrome P450 proteins were found within the identified QTL regions (Table 4). These genes may play a role in regulating coleoptile length based on existing literature.

## 4. Discussion

### 4.1. Coleoptile Length Variation in Oat

Frequent drought conditions resulting from climate change pose a significant challenge in many agricultural regions worldwide. Indeed, drought is the leading cause of reduced oat yield, surpassing all other factors in specific conditions [45]. To address this issue, the cultivation of oats with longer coleoptiles has emerged as a potential solution, as it can help alleviate emergence limitations and improve field establishment in water-limited areas. Previous studies in wheat have demonstrated that coleoptile length is primarily controlled by additive genetic factors, suggesting that rapid improvement in coleoptile length can be achieved by pyramiding of larger-effect QTLs in modern cultivars. Therefore, the first step is to identify QTLs associated with this trait. However, limited information is currently available on this topic in oat research. To fill this gap, we conducted a GWAS using a diverse panel comprising 243 oat varieties to generate valuable resources for the development of oats with longer coleoptiles.

Our results for phenotypic evaluation showed substantial variation in coleoptile length within the oat association panel, ranging from 4.66 to 8.76 cm, with a mean value of 6.55 cm (Table 2). These findings align with a previous study that reported a mean coleoptile length of 6.4 cm for four oat cultivars [19]. However, the coleoptile lengths in oat accessions used in our study are much longer than the 167 Turkish oats (coleoptile length values varied between 12.33 and 45.00 mm) reported by Koçak et al. [20]. The shorter coleoptile lengths observed in 167 Turkish oats could be attributed to the higher temperature (25 °C) applied during germination. In a previous study, Radford and Key [5] investigated the effects of germination temperature on the coleoptile lengths of 16 oat varieties and found that germination temperature over 25 °C significantly reduced the coleoptile length of all oat lines. Notably, oat accessions collected from the Mediterranean, Africa, and America exhibited longer coleoptile lengths than those from Asia and Europe, possibly indicating adaptation to different precipitation patterns. For example, wheat landraces in the Northern Spring Wheat Zone (NS) and the Northwestern Spring Wheat Zone (NWS) in China displayed longer coleoptile lengths compared to landraces from other agro-ecological zones [11]. Another study in wheat also showed that irrigated wheat cultivars have shorter coleoptile lengths compared to dryland cultivars [16]. Additionally, previous studies in wheat [15] revealed that the modern cultivars exhibited significantly shorter coleoptile lengths compared to the landraces. This was due to the widespread use of dwarfing genes, many of which had adverse effects on coleoptile length. In this study, however, there was no significant difference in coleoptile length between modern cultivars and landraces, suggesting that there is no specific focus or indirect selection for coleoptile length in any of the oat breeding programs. In addition, we observed a high heritability (*H*^2^ = 0.86) of coleoptile length in oats, consistent with findings in other cereal crops [11,16]. The significant phenotypic variation and heritability offer a strong basis for conducting a high-resolution GWAS targeting this trait.

### 4.2. Genomic Regions Associated with Coleoptile Length

This study utilized a comprehensive genome-wide association study (GWAS) with a diverse panel of oat accessions, genotyping a total of 26,196 single-nucleotide polymorphisms (SNPs). Our analysis successfully identified 34 SNPs that were significantly associated with coleoptile length in oats, corresponding to 32 distinct QTLs (Table S2). The impact of these QTLs on coleoptile length was relatively modest, explaining less than 10% of the phenotypic variance (*R*^2^). This finding aligns with previous studies in wheat [11,15] and barley [18], suggesting that coleoptile length is a complex trait regulated by multiple genetic factors. A previous study [20] identified five SNPs that were associated with coleoptile length in a panel of 167 Turkish oats. However, when comparing the 32 QTLs identified in this study with the reported SNPs, none of these QTLs were found to overlap with the known loci. The lack of overlap may be attributed to the differences in oat accessions, emphasizing the significance of including diverse populations when studying the genetic factors that influence coleoptile length. Furthermore, our results support previous research [15,40] by demonstrating an additive effect for coleoptile length among the identified QTLs. These findings highlight the potential of using these markers in combination to enhance oat coleoptile length.

### 4.3. Putative Candidate Genes for Coleoptile Length

Considerable studies have been conducted to identify genomic regions associated with coleoptile length in cereal crops, but only a few of these QTLs have been successfully cloned. In our study, we focused on identifying potential candidate genes within a 2.29 Mb region flanking the linkage disequilibrium (LD) region surrounding significant SNPs associated with oat coleoptile length. Through our analysis, we identified a total of 350 potential candidate genes. GO analysis revealed a significant enrichment of genes involved in various biological processes related to cell development, such as cell tip growth (GO:0009932), developmental cell growth (GO:0048588), and cell development (GO:0048468) (Figure S4). These findings were consistent with previous studies in barley [18] and wheat [40].

Phytohormones, such as gibberellins (GAs) and auxins, play a vital role in plant development and physiological processes and have been extensively studied in relation to coleoptile length. The dwarfing genes *RhtB1b* and *RhtD1b*, which are involved in the GA signaling pathway, have been identified as regulators of coleoptile length. These dwarfing genes act by reducing the signals of gibberellins, which are responsible for promoting cell elongation [46]. Consequently, the downregulation of the GA signaling pathway by *RhtB1b* and *RhtD1b* leads to a shorter coleoptile phenotype [15,47]. This reduction in gibberellin signaling leads to the suppression of cell elongation, ultimately affecting the overall length of the coleoptile. On the other hand, CRISPR/Cas9-induced *gibberellin (GA) 3-oxidase 1* (*GA3ox1*) increased wheat coleoptile length [48]. This manipulation of the GA pathway results in enhanced gibberellin production, leading to the promotion of cell elongation.

Auxins, another important group of phytohormones, directly induce cell elongation and significantly impact coleoptile length in grass species [49]. In our study, we identified a gene that encodes the putative auxin transporter-like protein 1 (AUX1) within the QTL *QCL-4D.2* (Table S3). AUX1, a member of the AUX/LAX family, mediates the transport of indole-3-acetic acid (IAA) in *Arabidopsis* [50]. In rice, mutants of *Osaux1* exhibited shorter coleoptiles compared to the background *japonica* variety “Dongjing”, which has long coleoptiles [43]. Additionally, the cytochrome P450 superfamily protein CYP87A3 has been characterized in rice as an auxin-induced gene specifically expressed in coleoptiles [42]. We found five putative cytochrome P450 proteins spanning the QTLs *QCL-1A.1* (3), *QCL-4C.1* (1), and *QCL-4D.2* (1) (Table 4), which may influence coleoptile length in oat. Further studies are necessary to elucidate the function and downstream targets of these genes in oat coleoptile development.

### 4.4. Limitations of This Study

Several potential limitations in this study should be considered. Firstly, the assessment of coleoptile length was carried out in a growth chamber, which may not represent the actual field conditions, since coleoptile length is significantly influenced by environmental factors such as temperature [5] and soil type [19]. Secondly, the markers used in this study were derived from GBS data, which may not cover the entire genome, particularly genomic regions with few enzyme-cut sites. This limitation could potentially hinder the effective identification of QTLs in these regions. Moreover, the high LD in oats imposes constraints on the mapping resolution through GWAS. Therefore, further studies are required to validate the detected markers and potential candidate genes before they are utilized for marker-assisted selection.

## 5. Conclusions

This study investigated the coleoptile lengths of 243 diverse oat accessions, revealing a significant range of variability. Six stable QTLs associated with coleoptile length were also identified through GWAS. The accessions with long coleoptile lengths can be used promptly for crosses with elite lines to produce new lines with improved coleoptile lengths. Specific crosses can also be made to create mapping populations, enabling fine mapping of the regions of interest. This will help identify diagnostic markers linked with long coleoptile length and ultimately clone the causal genes. Once cloned, the new genes can be transferred into elite germplasm using modern genome-assisted breeding strategies, such as gene cassettes and genome editing. As such, further characterization and validation of the detected loci are essential for the effective utilization of these results. Overall, this study emphasizes the genetic diversity present in oats and provides potential targets for enhancing coleoptile length.

## Figures and Tables

**Table 1:** Analysis of variance for coleoptile length.

Variables	df ^a^	Sum of Squares	Mean Squares	F-Values	Significance ^b^
Genotype	242	717.3	2.96	38.91	***
Experiment	1	0.9	0.90	11.77	***
G × E	242	32.0	0.13	1.74	***
Residuals	972	74.0	0.08		

^a^ df, degree of freedom; ^b^ ***, significant at the 0.001 level.

**Table 2:** Phenotypic variation and broad-sense heritability (H2) of coleoptile length.

Variables	Range (cm)	Mean (cm)	Standard Deviation (cm)	CV (%)	*H* ^2^
E1	4.70–8.75	6.57	0.71	10.77	
E2	4.66–8.68	6.52	0.73	11.13	
Total	4.66–8.75	6.55	0.75	11.45	0.86

**Table 3:** QTLs that were consistently detected in both experiments, including the designated name, linked SNP(s), chromosome location, physical position, alleles, −log_10_(*p*) value, and *R*^2^ value.

QTL	Marker	Chromosome	Position (Mb)	Allele *	E1			E2		
					−Log_10_ (*p*)	*R*^2^ (%)	Allelic Effects (%)	−Log_10_ (*p*)	*R*^2^ (%)	Allelic Effects (%)
*QCL-1A.1*	S1A_295026052	1A	295.03	A/**G**	3.72	8.11	4.05	3.82	8.35	3.09
*QCL-1D.1*	S1D_38632063	1D	38.63	A/**G**	3.54	7.64	5.57	3.16	6.83	5.27
*QCL-1D.2*	S1D_49143792	1D	49.14	G/**A**	3.37	7.77	4.03	3.51	8.05	3.54
*QCL-1D.5*	S1D_180046647	1D	180.05	G/**C**	3.36	7.09	6.05	3.05	6.44	4.30
	S1D_180046702	1D	180.05	G/**A**	3.43	7.42	8.84	3.09	6.43	6.54
*QCL-4C.1*	S4C_36128238	4C	36.13	A/**C**	4.38	10.07	9.05	3.25	7.30	8.79
*QCL-4D.2*	S4D_350378493	4D	350.38	C/**T**	3.53	8.07	9.26	3.31	7.69	7.86

* The favorable alleles are in bold type.

**Table 4:** Potential candidates related to coleoptile length according to literature.

QTL	Gene_ID	Distance (bp) to Significant SNP ^#^	Description	Literature
*QCL-1A.1*	*A.satnudSFS1A01G005205*	−1,936,883	Cytochrome P450 85A1-like	Chaban et al. [43]
	*A.satnudSFS1A01G005149*	1,517,948	Cytochrome P450 71A1	Chaban et al. [43]
	*A.satnudSFS1A01G005147*	1,682,924	Cytochrome P450 71A1	Chaban et al. [43]
*QCL-4C.1*	*A.satnudSFS4C01G001030*	1,742,832	Cytochrome P450 72A15	Chaban et al. [43]
*QCL-4D.2*	*A.satnudSFS4D01G002954*	1,583,082	Auxin transporter-like protein 1	Nghi et al. [44]
*QCL-4D.2*	*A.satnudSFS4D01G002963*	1,233,083	Cytochrome P450 CBH32594.1	Chaban et al. [43]

^#^ Physical distance from the SNP to the candidate gene. A negative value represents the upstream to the SNP.

## Data Availability

The data presented in this study are included in the article/Supplementary Materials; further inquiries can be directed to the corresponding authors.

## Supplementary Materials

The following supporting information can be downloaded at: https://www.mdpi.com/article/10.3390/genes15040411/s1, Figure S1: The first two principal components depicting the population structure of the Diverse Oat Panel. Blue dots represent accessions used in the present study; Figure S2: Correlations among experiments/replications. ***, significant at *p* < 0.001; Figure S3: Comparison of the Quantile-Quantile plots generated by GLM and MLM models; Figure S4: Gene ontology (GO) functional classification of 350 candidate genes located within the LD block on each side of the seven SNPs. Only significantly enriched GO terms were displayed. Q value rep-resent the BH adjusted *p* value; Table S1: List of materials used in the present study and their coleoptile length collected in two independent experiments; Table S2: List of QTLs that were significantly associated with coleoptile length in two independent experiments; Table S3: The genes inside the 2.29 Mb region on each side of the seven stable QTLs; Table S4: Significantly enriched GO terms associated with the candidate genes of coleoptile length.
