# Supplementary figures and images for: Genome-Wide Association Study Uncovers Genomic Regions Associated with Coleoptile Length in a Worldwide Collection of Oat
Source: Genes (Basel). 2024 Mar 26;15(4):411. doi: 10.3390/genes15040411 (PMC11049438; doi:10.3390/genes15040411)

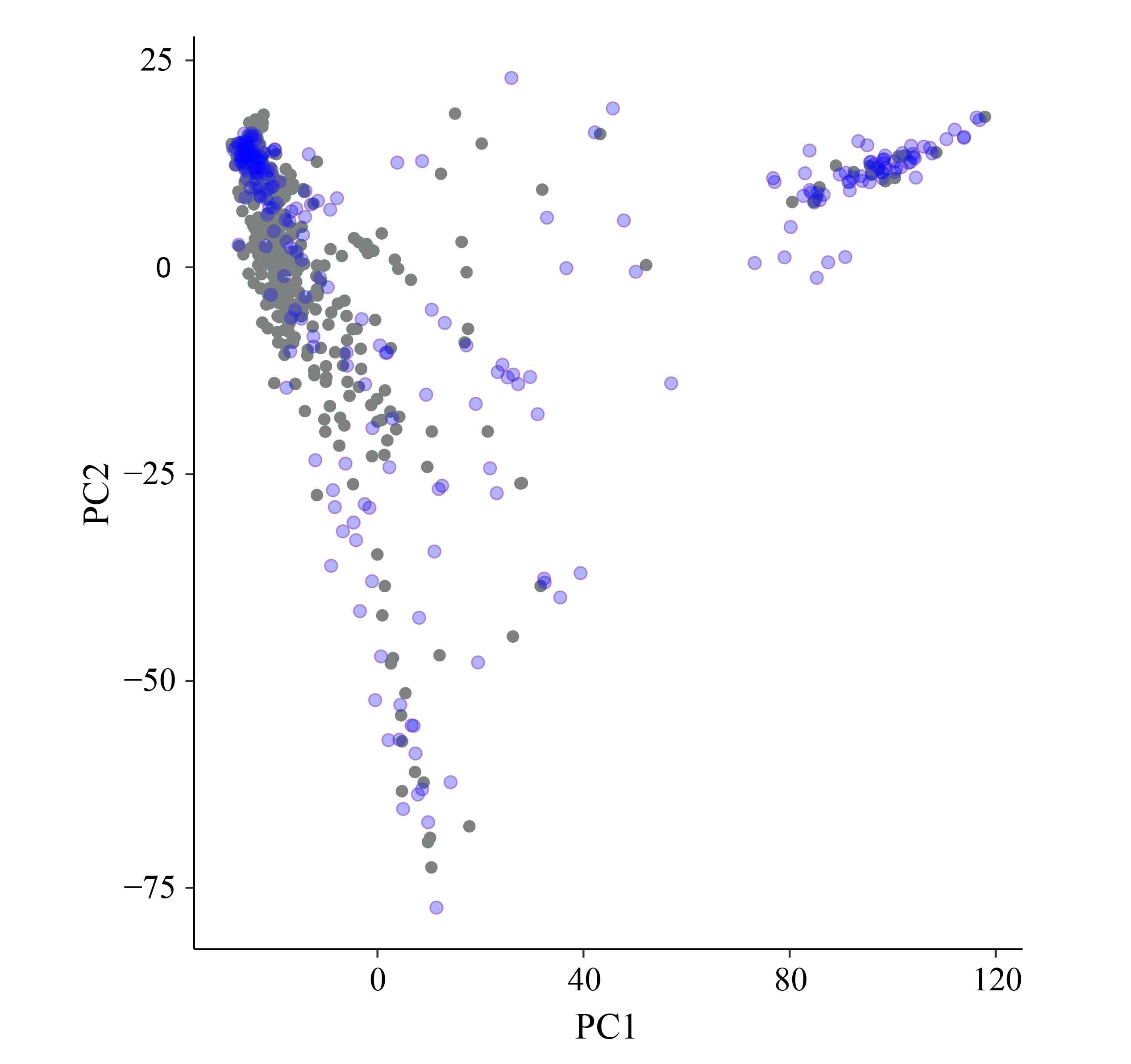

Supplement: Supplementary file 1 [file genes-15-00411-s001.zip › Figure S1.jpg]

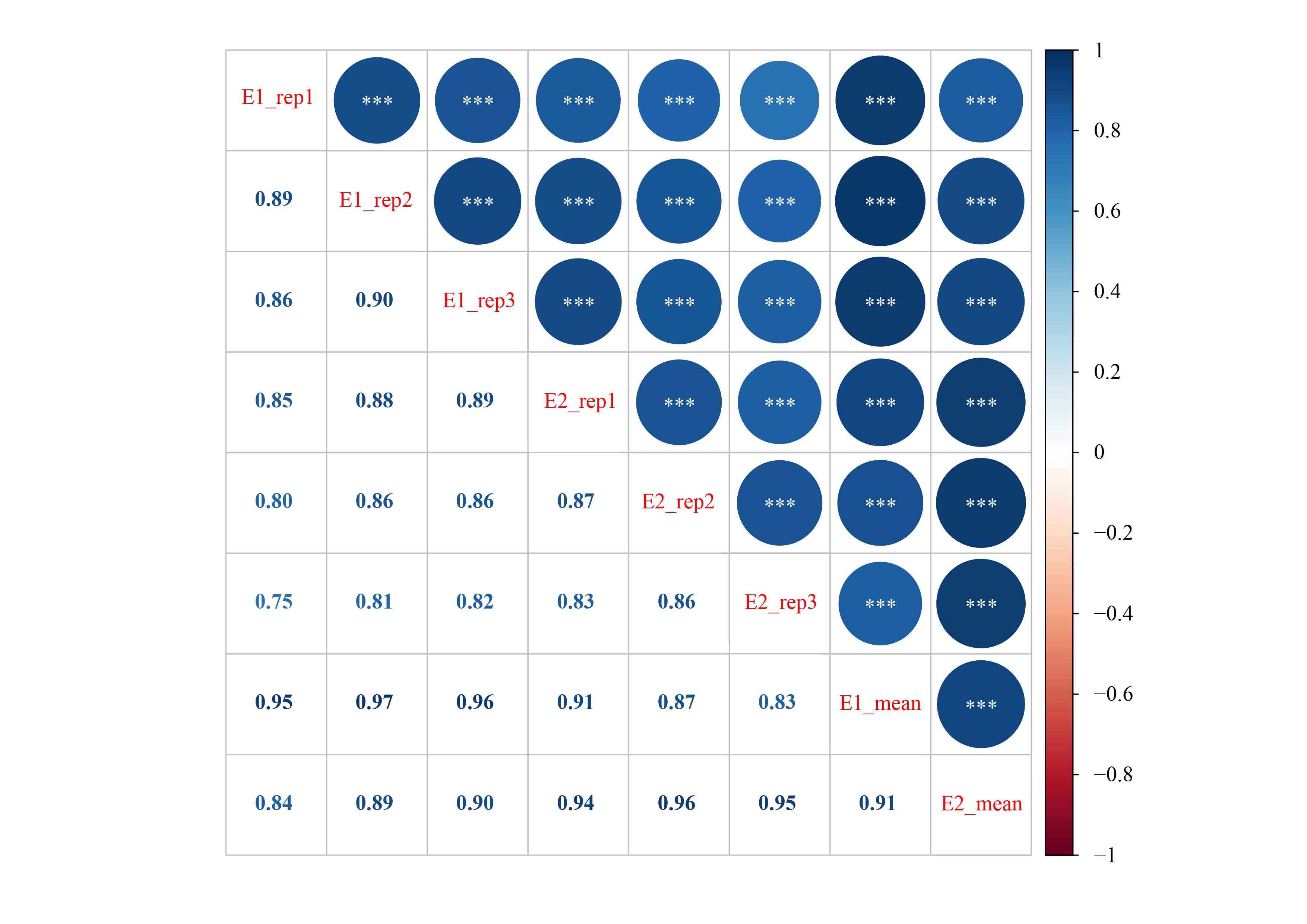

Supplement: Supplementary file 1 [file genes-15-00411-s001.zip › Figure S2.jpg]

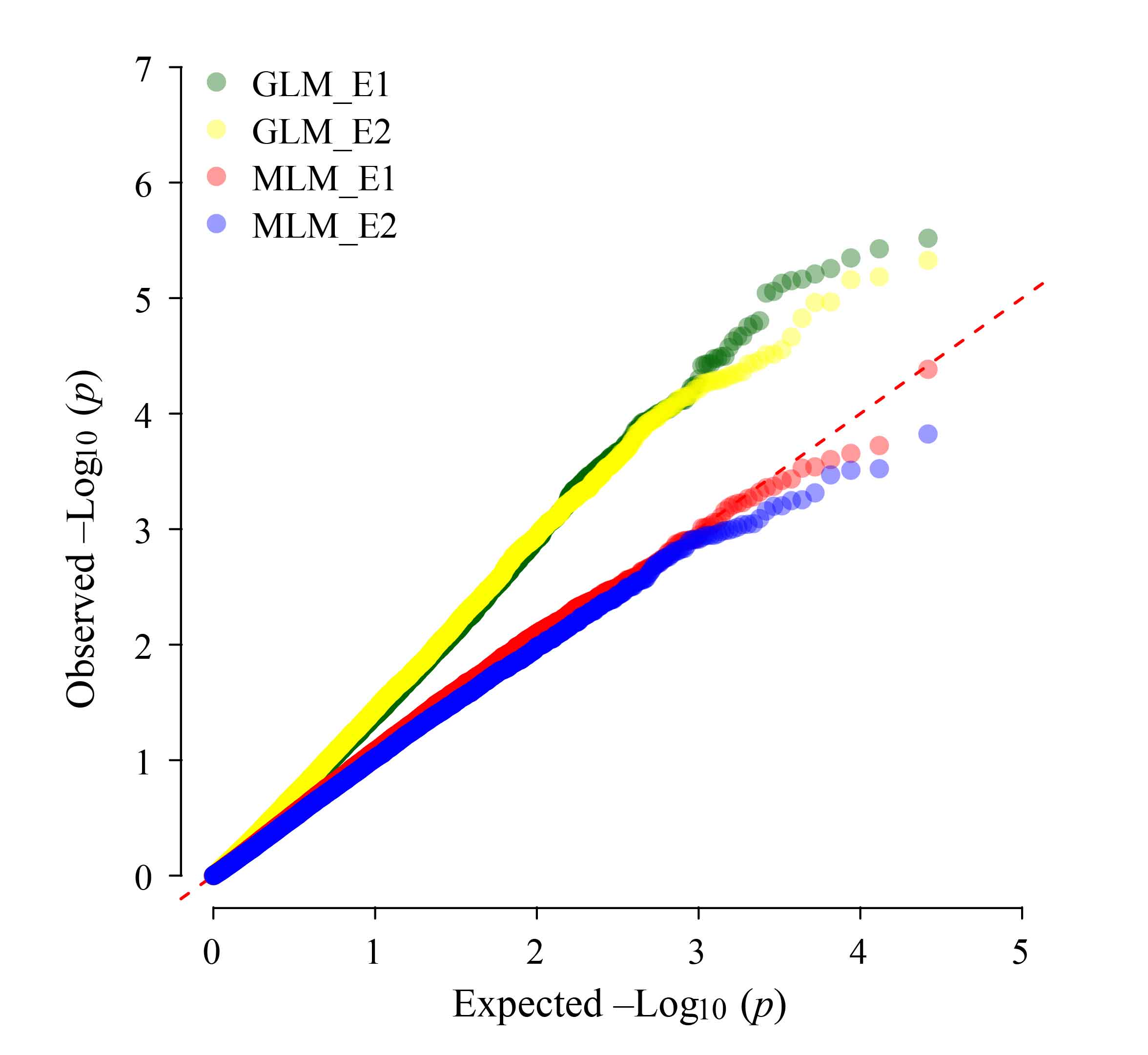

Supplement: Supplementary file 1 [file genes-15-00411-s001.zip › Figure S3.jpg]

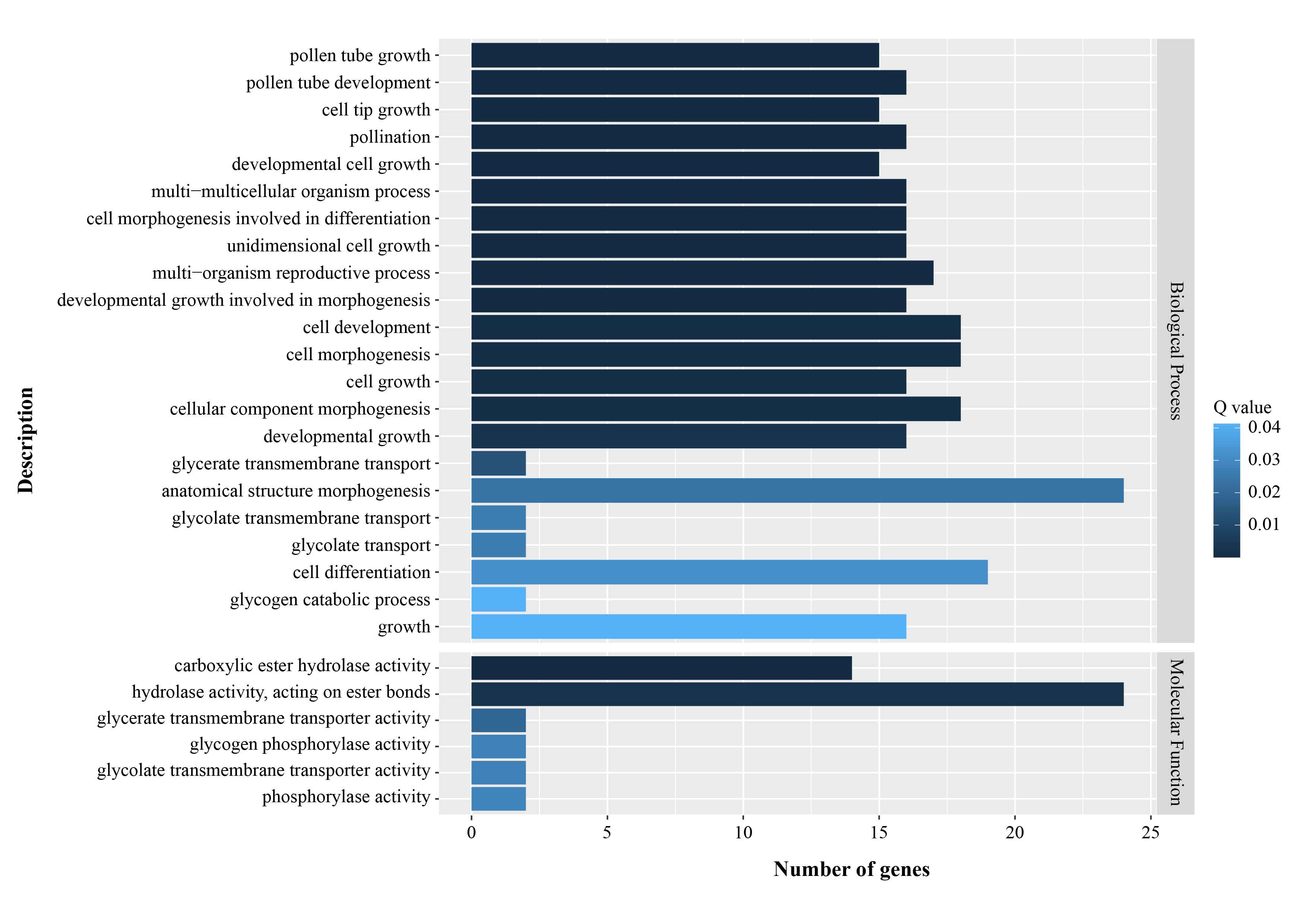

Supplement: Supplementary file 1 [file genes-15-00411-s001.zip › Figure S4.jpg]
